# Prognosis and Novel Drug Targets for Key lncRNAs of Epigenetic Modification in Colorectal Cancer

**DOI:** 10.1155/2023/6632205

**Published:** 2023-04-12

**Authors:** Peng Zhang, Tingting Zhang, Denggang Chen, Li Gong, Min Sun

**Affiliations:** ^1^Department of General Surgery, Taihe Hospital, Hubei University of Medicine, Shiyan, China; ^2^Department of Clinical Oncology, Taihe Hospital, Hubei University of Medicine, Shiyan, China; ^3^Department of Endocrinology, Taihe Hospital, Hubei University of Medicine, Shiyan, China

## Abstract

**Background:**

Colorectal cancer (CRC) has been the 3rd most commonly malignant tumor of the gastrointestinal tract in the world. 5-Methylcytosine (m^5^C) and long noncoding RNAs (lncRNAs) have an essential role in predicting the prognosis and immune response for CRC patients. Therefore, we built a m^5^C-related lncRNA (m^5^CRlncRNA) model to investigate the prognosis and treatment methods for CRC patients.

**Methods:**

Firstly, we secured the transcriptome and clinical data for CRC from The Cancer Genome Atlas (TCGA). Then, m^5^CRlncRNAs were recognized by coexpression analysis. Then, univariate Cox, least absolute shrinkage and selection operator (LASSO), and multivariate Cox regression analyses were utilized to build m^5^C-related prognostic characteristics. Besides, Kaplan-Meier analysis, ROC, PCA, *C*-index, enrichment analysis, and nomogram were performed to investigate the model. Additionally, immunotherapy responses and antitumor medicines were explored for CRC patients.

**Results:**

A total of 8 m^5^C-related lncRNAs (AC093157.1, LINC00513, AC025171.4, AC090948.2, ZEB1-AS1, AC109449.1, AC009041.3, and LINC02516) were adopted to construct a risk model to investigate survival and prognosis for CRC patients. CRC samples were separated into low- and high-risk groups, with the latter having a worse prognosis. The m^5^C-related lncRNA model helps us to better distinguish immunotherapy responses and IC50 of antitumor medicines in different groups of CRC patients.

**Conclusion:**

The research may give new perspectives on tailored therapy approaches as well as novel theories for forecasting the prognosis of CRC patients.

## 1. Introduction

In terms of cancer-related deaths, colorectal cancer (CRC) is the third most frequent malignant tumor worldwide [1]. The recent epidemiological surveys showed that CRC contributes to 10% of all diagnosed cancers and 9.4% of cancer-related deaths [2]. The high incidence and low survival rate of CRC imposed a heavy economic burden and enormous public health pressure on the government. At present, the main clinical treatment strategies for CRC include surgery, chemotherapy, and radiotherapy, but with poor prognosis, easy recurrence, and significant side effects [3]. In order to better understand CRC, it is urgently needed to select key CRC-related genes, elucidate the potential pathogenesis of CRC, and develop novel diagnostic and therapeutic strategies for CRC.

Numerous studies have found that RNA modifications in epigenetic changes are intimately related to the progression of different types of tumors [4, 5]. At present, more than 150 RNA modifications have been recognized, such as N1-methyladenosine (m^1^A), 5-methylcytosine (m^5^C), N6-methyladenosine (m^6^A), 7-methylguanosine (m^7^G), microRNA, and long noncoding RNA (lncRNA) [6]. With the in-depth study of RNA modification, m^5^C has received increasing attention from scholars around the world. As a widespread RNA modification of noncoding and coding RNAs, m^5^C has a crucial function in the regulation of physiological and pathological processes in the organism [7]. A study demonstrated that m^5^C regulators were linked to the occurrence and progression of cancer [8]. In bladder cancer, the m^5^C modification writer NSUN2 modulates HDGF expression in a m^5^C-dependent manner in order to promote cancer development [9].

lncRNA is the nonprotein-coding RNA fraction of over 200 nucleotides in length that cannot be translated into protein [10]. It has been shown that RNA methylation of lncRNAs could impact cancer progression [11]. With the advancement of sequencing technology, m^5^C was found to be extensively distributed in lncRNAs. However, the utility of m^5^C in lncRNAs is still uncertain. Therefore, identifying m^5^C-related lncRNAs (m^5^CRlncRNAs) in CRC pathogenesis may help provide a rational basis for targeted therapy and prognosis.

In this study, bioinformatics analysis was used to examine the potential contribution of m^5^CRlncRNAs to CRC. The Cancer Genome Atlas was used to obtain a database of m^5^C genes and lncRNAs (TCGA). Then, using Pearson's correlation analysis, we were able to identify the m^5^CRlncRNA. Additionally, a brand-new risk model for the m^5^CRlncRNA was developed to forecast overall survival (OS) in CRC patients. We also created a nomogram incorporating clinical data to predict the overall survival of CRC patients. Finally, we looked for the connection between immunotherapy responses.

## 2. Materials and Methods

### 2.1. Data Acquisition

TCGA database was utilized to retrieve RNA transcriptome data, relevant clinical information, and mutation data from CRC samples. We used the R package to process the downloaded files. To reduce statistical bias, we excluded CRC patients with absent OS values and short OS values (<30 days).

### 2.2. Identification m^5^C Genes and m^5^CRlncRNAs

Based on previous publications [12, 13], we extracted 17 m^5^C regulators from TCGA-CRC, including expression data on 11 writers, 2 readers, and 4 erasers (Supplementary Table 1). Then, we screened m^5^CRlncRNA by Pearson correlation analysis, and we derived 2,028 m^5^CRlncRNA. |*R*| > 0.5 and *p* < 0.001 were the threshold criteria.

### 2.3. Construction of a Risk Model

TCGA dataset was randomly distributed into a training set and a testing set (ratio: 0.7 : 0.3; sample: 355 : 148). We used the entire set to construct a m^5^CRlncRNA risk model, and the training set and testing set were employed to verify the risk model. No significant differences were found in the clinical features of CRC patients between the two sets (Table 1). We utilized univariate Cox analysis of the filtered 14 m^5^CRlncRNA in combination with CRC survival information (*p* < 0.01). Besides, we adopted the least absolute shrinkage and selection operator (LASSO) and Cox regression analyses to construct a risk assessment model that consisted of 8 m^5^CRlncRNAs via the R package “glmnet” [14]. According to median risk scores, the CRC patients were assigned to low- and high-risk groups [15]. And the risk score was calculated as follows: ∑_*i*=1_^*k*^*βiSi*.

### 2.4. Validation of the Risk Signature

By using the “survminer” and “survive” packages in the R programming language, Kaplan-Meier survival analysis was employed to compare the clinical outcomes of the two groups. The time-dependent receiver-operating characteristic curves (ROC) and the area under the curve (AUC) were employed to confirm the accuracy of the model. We also grouped patients according to clinical characteristics to assess the ability of the model to predict prognosis across clinical characteristics. Principal component analysis (PCA) was employed for effective dimension reduction, model recognition, and exploratory visualization of high-dimensional data of the whole gene expression profiles, m^5^C genes, m^5^CRlncRNAs, and a risk model on the basis of the expression patterns of the m^5^CRlncRNAs. A consistency index (*C*-index) was applied to determine the accuracy of the model compared to the traditional clinical features.

### 2.5. Construction of Predictive Nomogram

We developed a nomogram to predict the clinical features for the 1-, 3-, and 5-year OS of CRC patients via the R package of “rms.”.

### 2.6. Evaluation of Enrichment Analysis

A clusterProfiler R package was used to perform GO enrichment analysis and KEGG pathway analysis to explore possible biological functions. *p* < 0.05 indicated that the functional pathways had significant enrichment.

### 2.7. Assessment of the Prognostic Features in the Tumor Immune Microenvironment

Studying how the model interacts with the tumor microenvironment, we measured the infiltration values for TCGA-CRC dataset samples on the basis of these algorithms: XCELL [16], TIMER [17], QUANTISEQ [18], MCPCOUNTER [19], EPIC [20], CIBERSORT-ABS [21], and CIBERSORT [22]. Additionally, we adopted single-sample GSEA (ssGSEA) for scoring CRC-infiltrating immune cells to quantify the tumor-infiltrating immune cells between different groups. Furthermore, we also evaluated the immune checkpoint activation among different groups.

### 2.8. Investigation of Immunotherapy Response

The mutation data was assessed and summarized by the “maftools” of R package. Based on tumor-specific mutated genes, we calculated the tumor mutational burden (TMB). In addition, the tumor immune dysfunction and exclusion (TIDE) algorithm was performed to estimate the probability of an immunotherapeutic response.

### 2.9. Exploration of Antitumor Agents

To predict therapeutic response, the “pRRophetic” R package was employed to determine the half-maximal inhibitory concentration (IC50) of commonly used antitumor drugs in different risk groups.

## 3. Results

### 3.1. Screen of m^5^CRlncRNAs in CRC Patients

**Figure 1:**
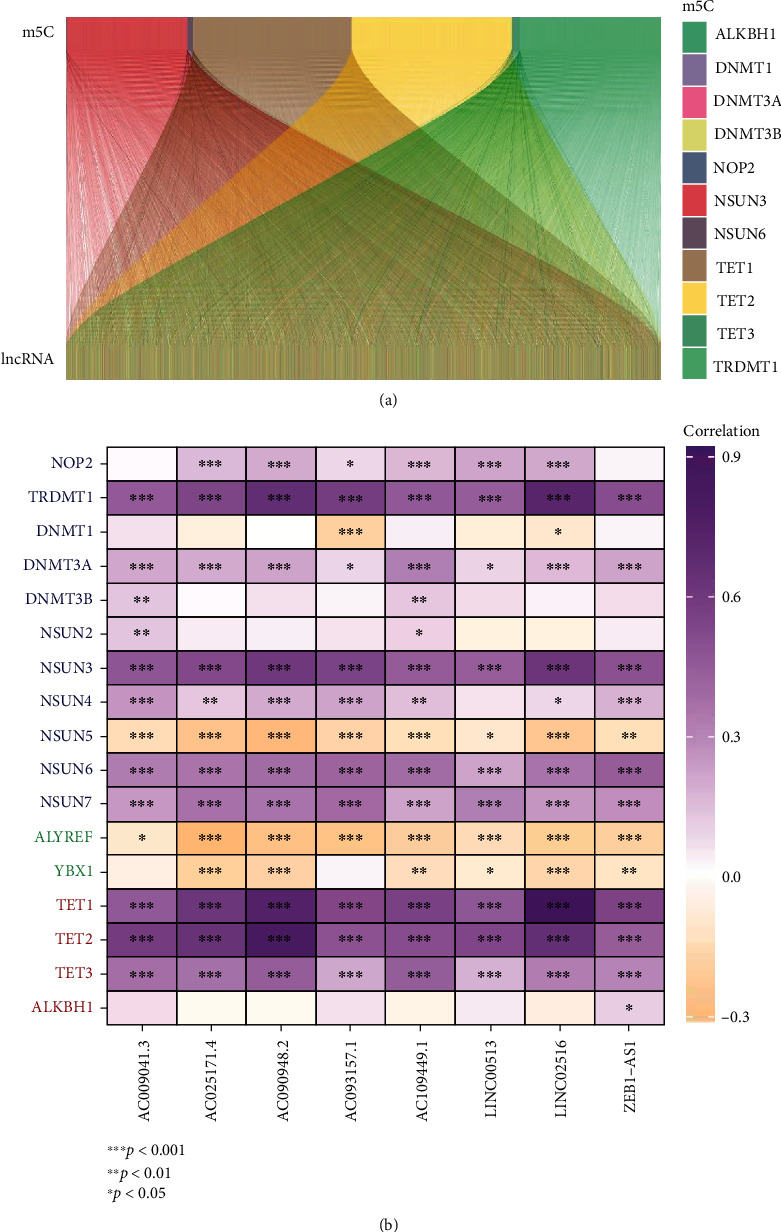
Identification of m^5^CRlncRNAs. (a) A Sankey plot for the network of m^5^C genes and associated m^5^CRlncRNAs. (b) Heatmap for correlation between 17 m^5^C genes and 8 m^5^CRlncRNAs.

A total of 17 m^5^C genes and 16,876 lncRNAs were selected from TCGA datasets. We found 2,028 lncRNAs that were strongly linked to one of the 17 m^5^C genes (|*R*| > 0.5 and *p* < 0.001) (Supplementary Table 2). As shown in Figure 1(a), the m^5^C-lncRNA expression network was visualized via the Sankey diagram.  Figure 1(b) depicts the relationship between m^5^C genes and m^5^CRlncRNAs in TCGA datasets.

### 3.2. Construction and Validation of a Risk Model

**Figure 2:**
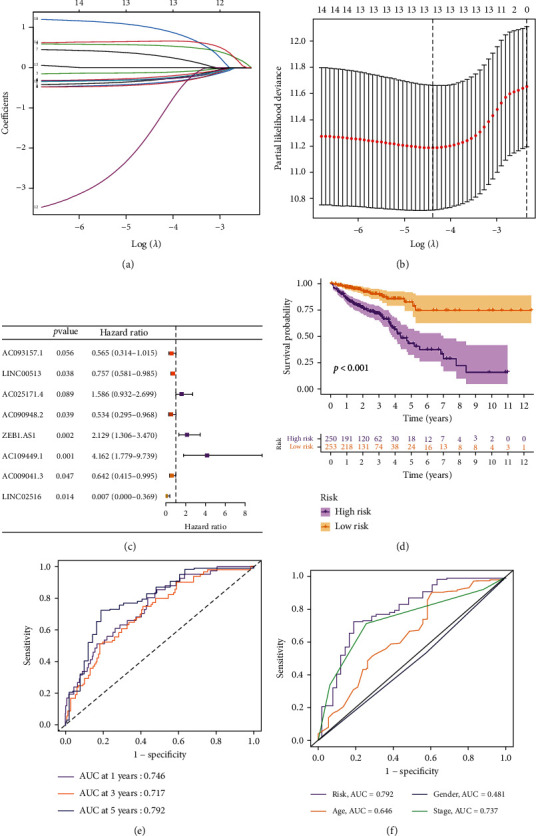
Construction of a risk signature for m^5^CRlncRNAs. (a, b) The LASSO regression algorithm to screen candidate m^5^CRlncRNAs. (c) Multivariate Cox regression analysis to develop a risk model. (d) Kaplan-Meier curves. (e) The 1-, 3-, and 5-year ROC curves of the entire set. (f) The 5-year ROC curves of the model and clinical characteristics.

We adopted univariate Cox regression analysis to select 14 prognostic m^5^CRlncRNAs (Supplementary Figure S1A). The LASSO-Cox regression algorithm was performed to construct the risk signature, including 8 m^5^CRlncRNAs (AC093157.1, LINC00513, AC025171.4, AC090948.2, ZEB1-AS1, AC109449.1, AC009041.3, and LINC02516) in CRC (Figures 2(a)–2(c)). In addition, Kaplan-Meier analysis revealed a significant difference between distinct groups (*p* < 0.05, Figure 2(d)). In Figure 2(e), the 1-, 3-, and 5-year AUC values were 0.746, 0.717, and 0.792, which demonstrated that CRC patients have a better prognosis. Furthermore, the AUC value of the signature was 0.792, which was notably higher than that of clinicopathological characteristics, including age (0.646), gender (0.481), and stage (0.737; Figure 2(f)). The Kaplan-Meier analyses and ROC curves of training set and testing set indicated that the prediction accuracy of the model is satisfactory (Supplementary Figure S1B-E).

**Figure 3:**
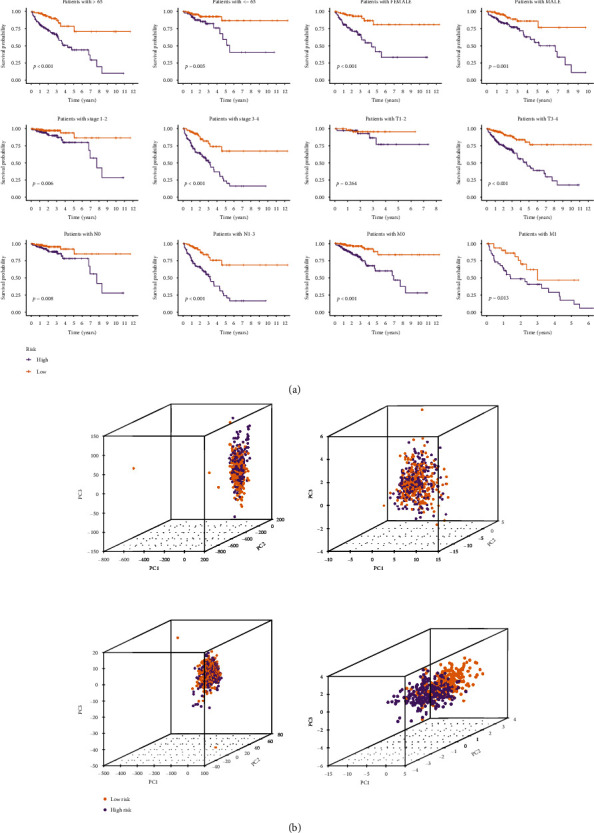
Validation of a risk signature. (a) Kaplan-Meier curves grouped by age, gender, clinical stage, T, N, or M. (b) PCA comparison on the basis of entire gene profiles, m^5^C genes, lncRNAs, and m^5^CRlncRNAs in TCGA entire set.

**Figure 4:**
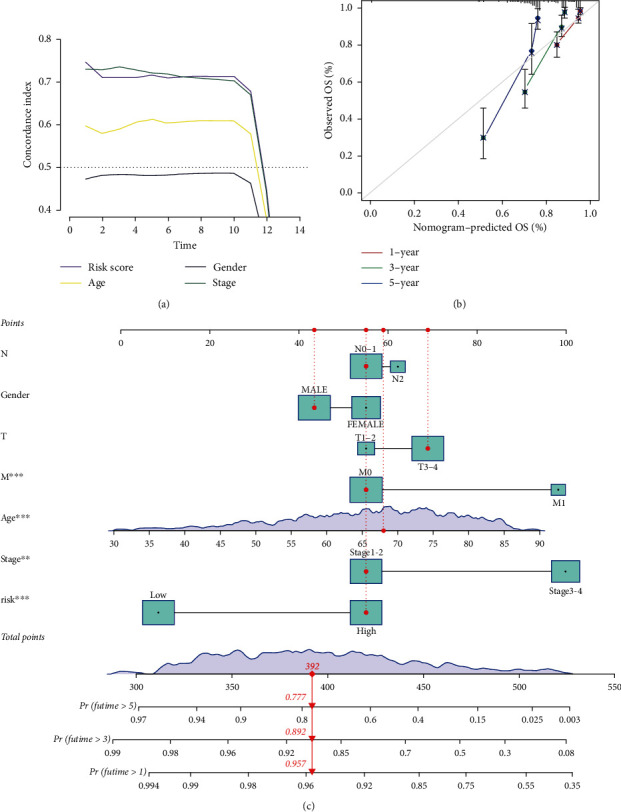
Evaluation of the m^5^CRlncRNAs model and development of a nomogram. (a) *C*-index of clinical characteristics and the model. (b) Calibration plot of a nomogram. (c) The nomogram predicts the ability of 1-, 3-, and 5-years OS rates of CRC patients.

Next, we studied the differences in clinical outcomes among distinct groups stratified by clinical characteristics. Kaplan-Meier survival analysis demonstrated that our model can be applied to a variety of clinical characteristics (Figure 3(a)). The PCA analysis showed that the distributions of the two groups were relatively dispersed, which indicated diverse groups had different distributions on the basis of the signature (Figure 3(b)). And the *C*-index of the model was superior to the clinicopathological features, indicating that this model could better predict the prognosis of CRC patients (Figure 4(a)).

### 3.3. Construction of Nomogram and Calibration in CRC Patients

As shown in Figure 4(b), the calibration curves of a nomogram revealed good accordance between the predicted 1-, 3-, and 5-year OS rates and actual observations. And the nomogram was established to demonstrate the superior predictive power of m^5^C compared to clinical features (Figure 4(c)).

### 3.4. Functional Enrichment Analysis

**Figure 5:**
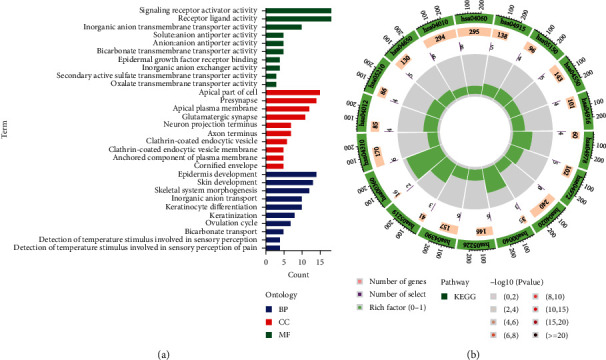
The enrichment analyses. (a) The GO analyses results. (b) The KEGG analysis results.

The GO analysis (Figure 5(a)) showed that the terms were mainly enriched in the signaling receptor activator activity and receptor ligand activity of biological processes (BP), the apical part of cell and presynapse of cellular component (CC), and the epidermis development and skin development of molecular function (MF) (Supplementary Table 3). The KEGG analysis showed that the terms were mainly enriched in hsa04978, hsa04972, hsa04020, hsa00040, hsa05226, and hsa04390 (Figure 5(b)) (Supplementary Table 4).

### 3.5. Evaluation of Tumor Immune Microenvironment

**Figure 6:**
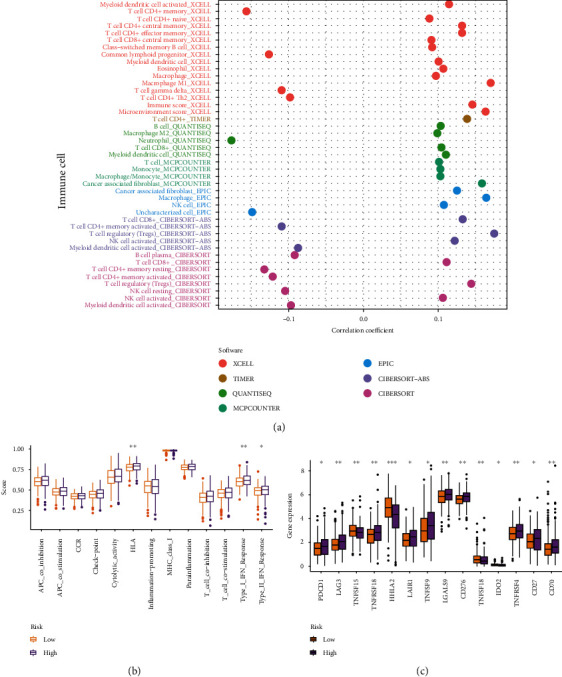
Immune analysis based on m^5^CRlncRNAs signature. (a) The immune cell bubble. (b) Immune functions scores between different groups. (c) Expression of immune checkpoint-related molecules.

To further explore whether the m^5^CRlncRNA was related to the TIME, we assessed the relationship between the signature and tumor-infiltrating immune cells. Significant correlations were noted between the abundance of these tumor-infiltrating immune cells and increased CRC risk (Figure 6(a)) (Supplementary Table 5). The ssGSEA results showed that HLA, type I IFN response, and type II IFN response of patients in the low-risk group were lower compared to high-risk group (*p* < 0.05, Figure 6(b)). We further investigated the immune checkpoints, and the results revealed significant differences in the distribution of immune checkpoint-related molecule expression among different groups. We examined the expression levels of 46 immune checkpoint genes, 14 of which differed in expression in the high- and low-risk groups. The immune checkpoints of TNFRSF15 and HHLA2 in the low-risk group were higher (*p* < 0.05) (Figure 6(c)). The above findings might imply that the low-risk group was more immunologically active and might be more sensitive to immunotherapy.

### 3.6. Evaluation of Immunotherapy

**Figure 7:**
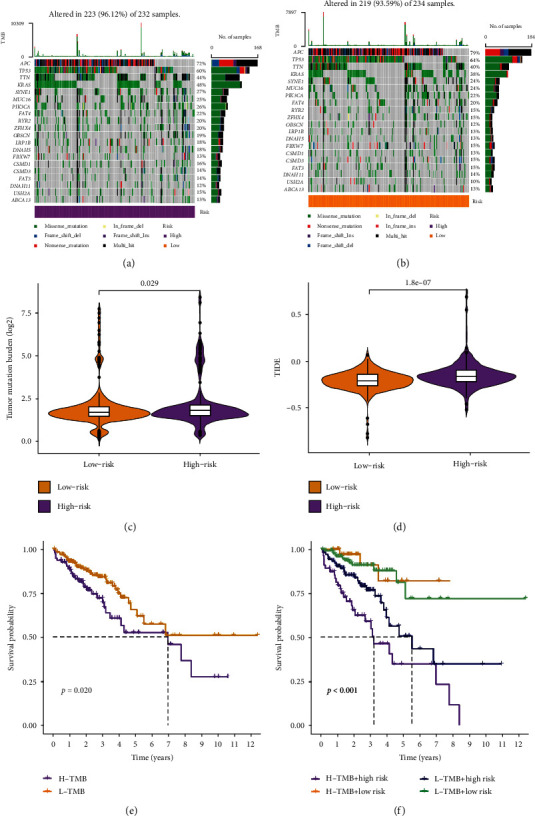
Assessment of immunotherapy response. (a, b) Waterfall plots revealed mutation information for genes with high mutation frequency. (c, d) TMB and TIDE of different groups. (e) Kaplan-Meier curve analysis different TMB groups. (f) Kaplan-Meier curve analysis of patient OS according to TMB and different risk groups.

On the basis of the somatic mutational data from TCGA, we calculated the mutation frequency among different groups. And the mutation frequencies of the different groups were depicted by the waterfall chart. It found that 223 of 232 (96.12%) CRC samples in the high-risk group and 219 of 234 (93.59%) CRC samples in the low-risk group displayed genetic mutations, and missense mutation was the most common variant classification. In addition, APC had high genetic alterations (72%), which was only lower than that of TP53 (60%) in the high-risk group (Figure 7(a)). APC had the highest genetic alterations (79%) (Figure 7(b)). Then, we evaluated the relationship between different groups and immunotherapy biomarkers. As exhibited in Figures 7(c) and 7(d), we observed that the high-risk group was more sensitive to immunotherapy, suggesting that the m^5^C-based classifier index may be a predictor of tumor mutation burden and TIDE. Finally, we used Kaplan-Meier curve analysis of patient OS based on TMB. As displayed in Figure 7(e), a significant difference was observed between patients in the high TMB and low TMB groups (*p* < 0.05). We further investigated whether the m^5^CRlncRNA model could predict OS outcomes greater than TMB alone. Compared with other groups, the low-risk group with high TMB had the best prognosis among those of the other three groups (*p* < 0.001) (Figure 7(f)). In summary, the signature of m^5^CRlncRNA has greater prognostic implications than that of TMB.

### 3.7. Selection Potential Antitumor Drugs by m^5^CRlncRNA Model

**Figure 8:**
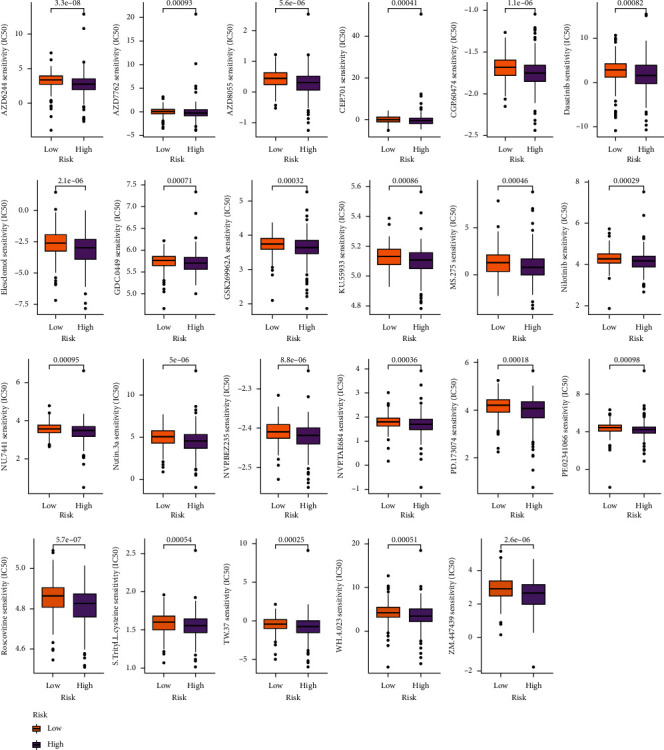
Selection of antitumor drugs.

To identify for potential drugs targeting the m^5^CRlncRNA model for the treatment of CRC patients, the pRRophetic algorithm was implemented to assess therapeutic efficacy according to the IC50 of each data. In addition, we noticed that the sensitivity of the two groups differed significantly for 23 compounds by predicting the potential therapeutic agents. As shown in Figure 8, we detected that the low-risk group was closely connected with chemotherapeutic agents with higher IC50, suggesting that low-risk patients were more responsive to chemotherapeutic drugs.

## 4. Discussion

Many experts and scholars have concentrated on exploring the pathogenesis and treatment strategies of CRC in recent years [23]. Despite the fact that surgery, chemotherapy, radiotherapy, and targeted therapy were used for CRC patients, treatment outcomes were poor, and 5-year survival rates were low [24]. In recent years, research has demonstrated that cancer patients with different clinical characteristics and subgroups are likely to have a different prognosis and response to treatment [25, 26]. Thus, it is vital to investigate effective and personalized treatment options for the prognosis and management of CRC.

Firstly, we downloaded the lncRNA data of CRC patients from TCGA database. According to univariate Cox, LASSO, and multivariate Cox regression analysis, 8 m^5^CRlncRNAs (AC093157.1, LINC00513, AC025171.4, AC090948.2, ZEB1-AS1, AC109449.1, AC009041.3, and LINC02516) were determined to be significant prognostic factors to explore the prognostic function in CRC. In recent years, lncRNAs have been linked to cancer survival and development in many studies [27, 28]. And ZEB1-AS1 was found to be a tumor-related lncRNA prognostic factor in CRC [29]. In addition, a study revealed that regulation of LINC00513 lncRNA expression can affect disease susceptibility in systemic lupus erythematosus [30]. Besides, other lncRNAs were screened for the first time as prognostic markers for CRC. Based on 8 m^5^CRlncRNAs, we built a risk assessment model to further investigate the association between m^5^CRlncRNA and CRC. Next, CRC patients were divided into different risk groups based on median scores, and the high-risk group had a lower clinical survival rate. There were similar results found in the analysis of subgroups categorized by gender, age, and tumor stage. Additionally, PCA analysis also supported the grouping ability of m^5^C. As part of the present study, we developed a nomogram with clinical characteristics and m^5^CRlncRNAs. These results suggest that OS was shorter in the high-risk group, with better concordance between 1-year, 3-year, and 5-year observation and predicted OS rates.

Furthermore, we investigated the associations between risk groups and TMB and TIME. The TMB is regarded as the total number of somatic cell-encoded mutations that lead to neoantigens being generated that trigger antitumor immunity [31]. A large number of studies have proven that TMB is a powerful biomarker for predicting the efficacy of checkpoint inhibitors in cancer [32, 33]. In the high-risk group, TMB appeared to be significantly higher. Additionally, TIDE is a computational framework for simulating tumor immune evasion and is usually applied to forecast the effects of immune checkpoint inhibition therapy [34]. Our results demonstrated that the low-risk group was predicted to have a superior response to immunotherapy. To probe the therapeutic potential of the identified m^5^CRlncRNAs for CRC patients, we analyzed their sensitivity to different drugs. And we discovered that the low-risk group was significantly related to chemotherapeutic agents with higher IC50. Altogether, the above results help us to further predict the prognosis of CRC and elucidate the molecular biological mechanism between m^5^CRlncRNAs and CRC.

However, there are several issues with the research. First off, we lack external datasets to validate the prediction accuracy of the risk model since there are not any lncRNA-related CRC datasets in the Gene Expression Omnibus (GEO) database. Second, because of the study's limited sample size, there could be some bias in the data analysis. Thirdly, there is no experimental validation of analytical findings in the research to demonstrate the use of the risk model in clinical treatment. Therefore, we will try to verify the validity of the signature utilizing animal and cellular tests.

Overall, our study did provide not only new insights into individualized treatment strategies for patients with CRC but also new ideas for predicting the prognosis of these patients. In addition, this study might contribute to further exploring the biological functions of m^5^C-regulated lncRNAs.

## Figures and Tables

**Table 1:** The clinical characteristics of the different sets.

Covariates	Type	Total set	Training set	Testing set	*p* value
Age	≤65	227 (45.13%)	161 (45.35%)	66 (44.59%)	0.9543
	>65	276 (54.87%)	194 (54.65%)	82 (55.41%)	

Gender	Female	225 (44.73%)	157 (44.23%)	68 (45.95%)	0.7985
	Male	278 (55.27%)	198 (55.77%)	80 (54.05%)	

Race	Asian	9 (1.79%)	6 (1.69%)	3 (2.03%)	0.9398
	Black or African American	246 (48.91%)	175 (49.3%)	71 (47.97%)	
	White	248 (49.3%)	174 (49.01%)	74 (50%)	

Radiation	No	466 (92.64%)	329 (92.68%)	137 (92.57%)	1
	Yes	37 (7.36%)	26 (7.32%)	11 (7.43%)	

Pharmaceutical therapy	No	301 (59.84%)	212 (59.72%)	89 (60.14%)	1
	Yes	202 (40.16%)	143 (40.28%)	59 (39.86%)	

Pathological stage	Stage I	91 (18.09%)	61 (17.18%)	30 (20.27%)	0.7053
	Stage II	180 (35.79%)	129 (36.34%)	51 (34.46%)	
	Stage III	155 (30.82%)	113 (31.83%)	42 (28.38%)	
	Stage IV	77 (15.31%)	52 (14.65%)	25 (16.89%)	

Stage T	T1	17 (3.38%)	12 (3.38%)	5 (3.38%)	0.0901
	T2	92 (18.29%)	60 (16.9%)	32 (21.62%)	
	T3	343 (68.19%)	253 (71.27%)	90 (60.81%)	
	T4	50 (9.94%)	30 (8.45%)	20 (13.51%)	
	Tis	1 (0.2%)	0 (0%)	1 (0.68%)	

Stage M	M0	377 (74.95%)	267 (75.21%)	110 (74.32%)	0.7588
	M1	77 (15.31%)	52 (14.65%)	25 (16.89%)	
	Mx	49 (9.74%)	36 (10.14%)	13 (8.78%)	

Stage N	N0	283 (56.26%)	200 (56.34%)	83 (56.08%)	0.7087
	N1	126 (25.05%)	86 (24.23%)	40 (27.03%)	
	N2	92 (18.29%)	67 (18.87%)	25 (16.89%)	
	Nx	2 (0.4%)	2 (0.56%)	0 (0%)	

Status	Alive	403 (80.12%)	284 (80%)	119 (80.41%)	1
	Dead	100 (19.88%)	71 (20%)	29 (19.59%)	

No: the patient have not receive the treatment; Tis: carcinoma in situ; Mx: unknown M stage; Nx: unknown N stage.

## Data Availability

The original contributions presented in the study are included in the article/Supplementary Material. Further inquiries can be directed to the corresponding author.

## Supplementary Materials

Supplementary 1 Supplementary Figure S1: (a) the prognostic m^5^CRlncRNAs by univariate Cox regression analysis. (b–e) Kaplan-Meier curves and the 1-, 3-, and 5-year ROC curves of the training and testing sets.

Supplementary 2 Supplementary Table S1: the list of m^5^C genes.

Supplementary 3 Supplementary Table S2: the details of m^5^CRlncRNAs.

Supplementary 4 Supplementary Table S3: the details of GO enrichment analysis.

Supplementary 5 Supplementary Table S4: the details of KEGG enrichment analysis.

Supplementary 6 Supplementary Table S5: the abundance of these tumor-infiltrating immune cells and increased CRC risk.

## References

[1] Zhang W., Peng C., Shen X. (2021). A bioactive compound from Sanguisorba officinalis L. inhibits cell proliferation and induces cell death in 5-fluorouracil-sensitive/resistant colorectal cancer cells. *Molecules*.

[2] Xi Y., Xu P. (2021). Global colorectal cancer burden in 2020 and projections to 2040. *Translational Oncology*.

[3] Zhang W., Sang S., Peng C. (2022). Network pharmacology and transcriptomic sequencing analyses reveal the molecular mechanism of Sanguisorba officinalis against colorectal cancer. *Frontiers in Oncology*.

[4] Bai M., Sun C. (2022). M5C-related lncRNA predicts lung adenocarcinoma and tumor microenvironment remodeling: computational biology and basic science. *Frontiers in Cell and Development Biology*.

[5] Shao D., Li Y., Wu J. (2022). An m6A/m5C/m1A/m7G-related long non-coding RNA signature to predict prognosis and immune features of glioma. *Frontiers in Genetics*.

[6] Barbieri I., Kouzarides T. (2020). Role of RNA modifications in cancer. *Nature Reviews Cancer*.

[7] Li M., Tao Z., Zhao Y. (2022). 5-Methylcytosine RNA methyltransferases and their potential roles in cancer. *Journal of Translational Medicine*.

[8] Liu X., Wang D., Han S. (2022). Signature of m5C-related lncRNA for prognostic prediction and immune responses in pancreatic cancer. *Journal of Oncology*.

[9] Chen X., Li A., Sun B. F. (2019). 5-Methylcytosine promotes pathogenesis of bladder cancer through stabilizing mRNAs. *Nature Cell Biology*.

[10] Liu L., Li X., Shi Y., Chen H. (2021). Long noncoding RNA DLGAP1-AS1 promotes the progression of glioma by regulating the miR-1297/EZH2 axis. *Aging (Albany NY)*.

[11] Pan J., Huang Z., Xu Y. (2021). m5C-related lncRNAs predict overall survival of patients and regulate the tumor immune microenvironment in lung adenocarcinoma. *Frontiers in Cell and Development Biology*.

[12] Zhang J., Wang N., Wu J. (2022). 5-Methylcytosine related lncRNAs reveal immune characteristics, predict prognosis and oncology treatment outcome in lower-grade gliomas. *Frontiers in Immunology*.

[13] Zhang H., Xu P., Song Y. (2021). Machine-learning-based m5C score for the prognosis diagnosis of osteosarcoma. *Journal of Oncology*.

[14] Wang H., Cui J., Yu J., Huang J., Li M. (2022). Identification of fatty acid metabolism-related lncRNAs as biomarkers for clinical prognosis and immunotherapy response in patients with lung adenocarcinoma. *Frontiers in Genetics*.

[15] Hong W., Liang L., Gu Y. (2020). Immune-related lncRNA to construct novel signature and predict the immune landscape of human hepatocellular carcinoma. *Molecular Therapy-Nucleic Acids*.

[16] Aran D., Hu Z., Butte A. J. (2017). xCell: digitally portraying the tissue cellular heterogeneity landscape. *Genome Biology*.

[17] Li T., Fan J., Wang B. (2017). TIMER: a web server for comprehensive analysis of tumor-infiltrating immune cells. *Cancer Research*.

[18] Finotello F., Mayer C., Plattner C. (2019). Molecular and pharmacological modulators of the tumor immune contexture revealed by deconvolution of RNA-seq data. *Genome Medicine*.

[19] Dienstmann R., Villacampa G., Sveen A. (2019). Relative contribution of clinicopathological variables, genomic markers, transcriptomic subtyping and microenvironment features for outcome prediction in stage II/III colorectal cancer. *Annals of Oncology: Official Journal of the European Society for Medical Oncology*.

[20] Racle J., de Jonge K., Baumgaertner P., Speiser D. E., Gfeller D. (2017). Simultaneous enumeration of cancer and immune cell types from bulk tumor gene expression data. *eLife*.

[21] Tamminga M., Hiltermann T. J. N., Schuuring E., Timens W., Fehrmann R. S., Groen H. J. (2020). Immune microenvironment composition in non-small cell lung cancer and its association with survival. *Clinical & Translational Immunology*.

[22] Newman A. M., Liu C. L., Green M. R. (2015). Robust enumeration of cell subsets from tissue expression profiles. *Nature Methods*.

[23] Jia W., Yuan L., Ni H., Xu B., Zhao P. (2021). Prognostic value of platelet-to-lymphocyte ratio, neutrophil-to-lymphocyte ratio, and lymphocyte-to-white blood cell ratio in colorectal cancer patients who received neoadjuvant chemotherapy. *Technology in Cancer Research & Treatment*.

[24] Wang X., Li T. (2020). Development of a 15-gene signature for predicting prognosis in advanced colorectal cancer. *Bioengineered*.

[25] Xu A., Wang Q., Lin T. (2020). Low-frequency magnetic fields (LF-MFs) inhibit proliferation by triggering apoptosis and altering cell cycle distribution in breast cancer cells. *International Journal of Molecular Sciences*.

[26] Ping L., Zhang K., Ou X., Qiu X., Xiao X. (2021). A novel pyroptosis-associated long non-coding RNA signature predicts prognosis and tumor immune microenvironment of patients with breast cancer. *Frontiers in Cell and Development Biology*.

[27] Wang Z., Chen J., Sun F. (2022). lncRNA CRLM1 inhibits apoptosis and promotes metastasis through transcriptional regulation cooperated with hnRNPK in colorectal cancer. *Cell & Bioscience*.

[28] Samadi P., Soleimani M., Nouri F., Rahbarizadeh F., Najafi R., Jalali A. (2022). An integrative transcriptome analysis reveals potential predictive, prognostic biomarkers and therapeutic targets in colorectal cancer. *BMC Cancer*.

[29] Ruan L., Chen W., Zhao X., Fang N., Li T. (2022). Predictive potentials of ZEB1-AS1 in colorectal cancer prognosis and their correlation with immunotherapy. *Journal of Oncology*.

[30] Xue Z., Cui C., Liao Z. (2018). Identification of lncRNA Linc00513 containing lupus-associated genetic variants as a novel regulator of interferon signaling pathway. *Frontiers in Immunology*.

[31] Bai Y., Xu Y., Wang X. (2020). Whole exome sequencing of lung adenocarcinoma and lung squamous cell carcinoma in one individual: a case report. *Thoracic Cancer*.

[32] Li H., Liu L., Huang T. (2021). Establishment of a novel ferroptosis-related lncRNA pair prognostic model in colon adenocarcinoma. *Aging (Albany NY)*.

[33] Jiang Z., Yin W., Zhu H. (2021). METTL7B is a novel prognostic biomarker of lower-grade glioma based on pan-cancer analysis. *Cancer Cell International*.

[34] Lv M. Y., Wang W., Zhong M. E. (2022). DNA repair-related gene signature in predicting prognosis of colorectal cancer patients. *Frontiers in Genetics*.

