# Supplementary figures and images for: Prognosis and Novel Drug Targets for Key lncRNAs of Epigenetic Modification in Colorectal Cancer
Source: Mediators Inflamm. 2023 Apr 12;2023:6632205. doi: 10.1155/2023/6632205 (PMC10116225; doi:10.1155/2023/6632205)

A

|            | pvalue | Hazard ratio        |
|------------|--------|---------------------|
| AC093157.1 | 0.008  | 0.467(0.266–0.818)  |
| LINC00513  | 0.004  | 0.683(0.529–0.883)  |
| AP002336.2 | 0.006  | 0.520(0.327–0.827)  |
| AC012313.5 | 0.005  | 0.331(0.152–0.717)  |
| AC011815.1 | 0.005  | 0.391(0.201–0.758)  |
| AC008121.2 | 0.007  | 0.359(0.170–0.761)  |
| AC025171.4 | 0.007  | 1.798(1.170–2.761)  |
| AC090948.2 | 0.006  | 0.526(0.331–0.836)  |
| ZEB1-AS1   | <0.001 | 2.503(1.615–3.881)  |
| AC109449.1 | 0.004  | 2.987(1.410–6.326)  |
| AC009041.3 | 0.007  | 0.606(0.420–0.872)  |
| LINC02516  | 0.002  | 0.002(0.000–0.100)  |
| PCAT1      | 0.008  | 0.452(0.251–0.815)  |
| AC011997.1 | <0.001 | 5.465(2.212–13.500) |

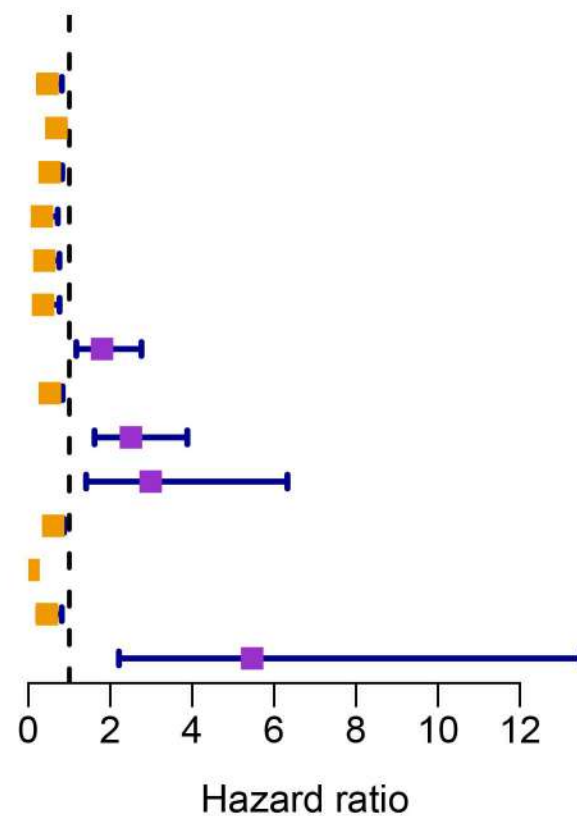

B

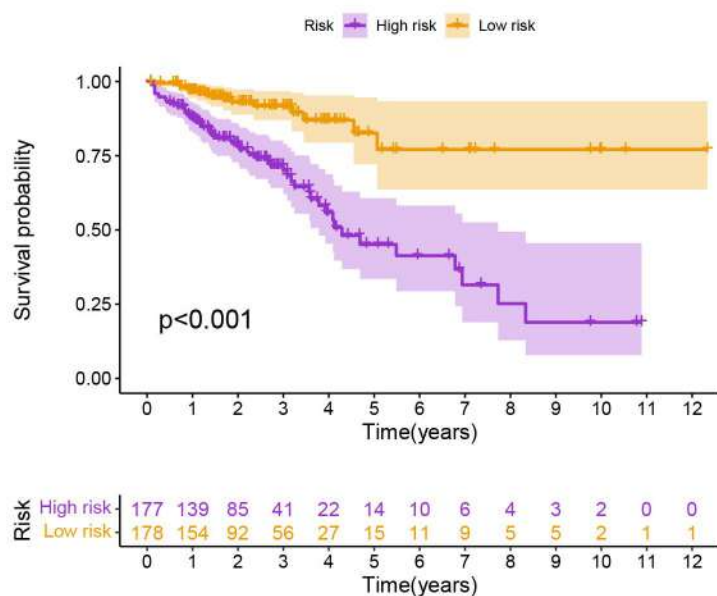

C

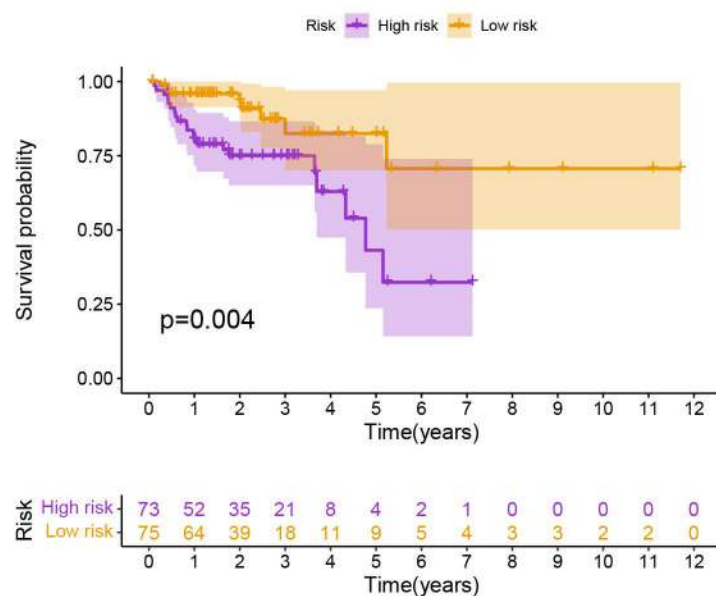

D

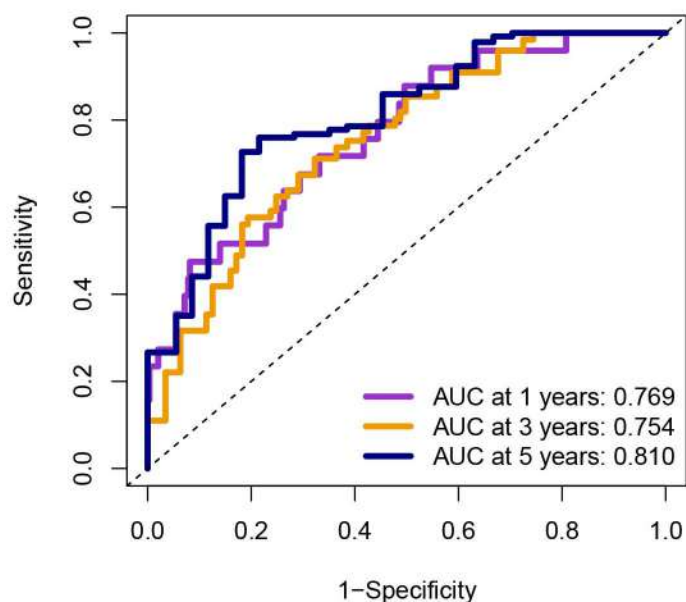

E

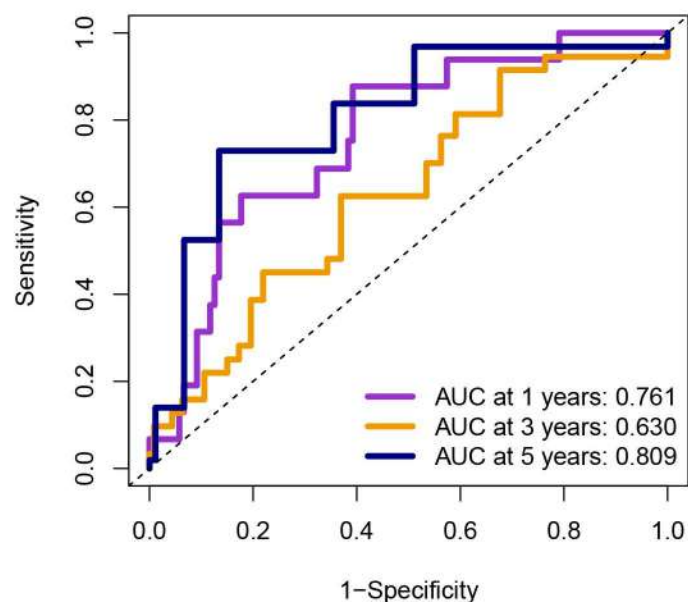

Supplement: Supplementary 1 — Supplementary Figure S1: (a) the prognostic m5CRlncRNAs by univariate Cox regression analysis. (b–e) Kaplan-Meier curves and the 1-, 3-, and 5-year ROC curves of the training and testing sets. [file 6632205.f1.pdf]
